# A case of early reperfusion of celiac artery for type A acute aortic dissection with malperfusion

**DOI:** 10.1186/s44215-023-00037-2

**Published:** 2023-05-02

**Authors:** Ryo Izubuchi, Keiji Uchida, Shota Yasuda, Tomoki Cho, Atsushi Matsumoto, Shotaro Kaneko, Makoto Ikematsu, Sho Kakuta, Sousuke Yamamoto, Makoto Ogata

**Affiliations:** Cardiovascular Center, Yokohama City University Medical Center, 4-57 Urafune-Cho, Minami-Ku, Yokohama, 232-0024 Japan

**Keywords:** Celiac malperfusion, Acute aortic dissection, Endovascular treatment

## Abstract

Malperfusion of the celiac artery alone, with acute aortic dissection, rarely requires early intervention. A 57-year-old woman had thrombosed type A acute aortic dissection with celiac malperfusion, for which total arch replacement was performed after percutaneous balloon angioplasty of the celiac artery. The endovascular treatment minimized extensive hepatic infarction, which saved the patient's life.

## Background

Visceral malperfusion associated with aortic dissection (AD) is a serious and sometimes life-threatening complication. However, a few cases of malperfusion of the celiac artery alone require early intervention. In this report, we describe a rare case of type A acute aortic dissection in which reperfusion of the celiac artery was performed before central repair.

## Case presentation

**Fig. 1:**
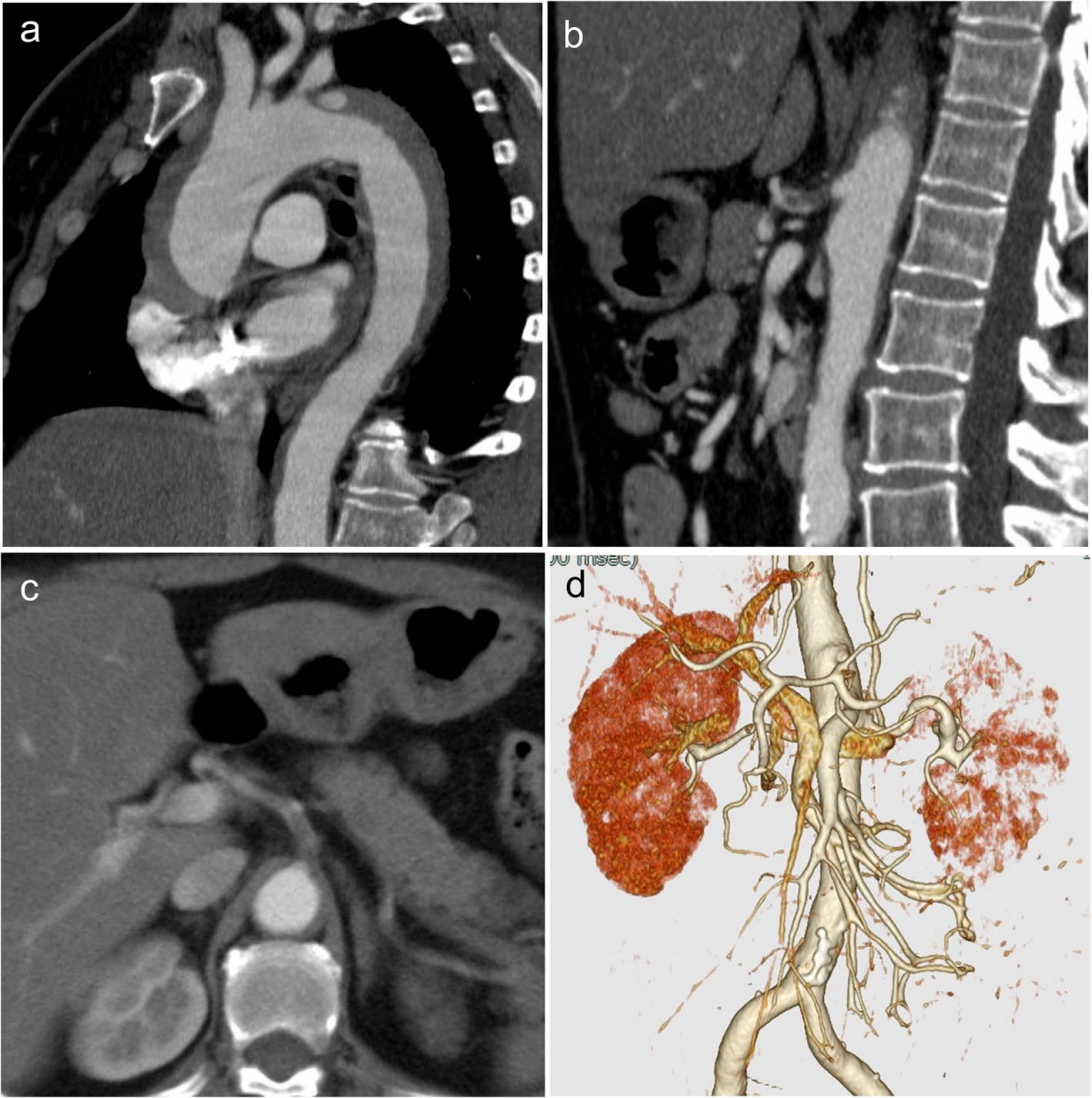
**a**, **b**, **c**, and **d** Preoperative contrast-enhanced CT. **a** Thrombosed type A acute aortic dissection, **b** and **c** severely stenotic celiac artery, and **d** poor collateral pathways of the celiac artery and superior mesenteric artery

A 57-year-old woman was transferred to the emergency room with sudden onset of back and severe abdominal pain. Contrast-enhanced computed tomography (CT) showed a thrombosed type A acute aortic dissection, and an ulcer-like projection was observed at the proximal descending aorta (Fig. 1a). There were no abnormal findings in the neck vessels, superior mesenteric artery, or bilateral renal arteries. However, the dissection extended to the celiac artery, which was severely stenotic or occluded (Fig. 1b and c). In the arterial phase, the hepatic and splenic arteries were well defined, but the collateral pathways of the celiac and superior mesenteric arteries were unclear (Fig. 1d). Due to the persistent severe abdominal pain, short lesion of the celiac artery, which seemed relatively easy to treat endovascularly, and the absence of circulatory collapse due to aortic dissection, endovascular treatment of the celiac artery was performed as the first line of treatment in this case. Percutaneous balloon angioplasty seemed to improve the blood flow in the celiac artery, and the patient’s abdominal pain was relieved. Unfortunately, a large-diameter short stent was unavailable then, as the surgery was performed in the middle of the night. After that, a total arch replacement was performed.

**Fig. 2:**
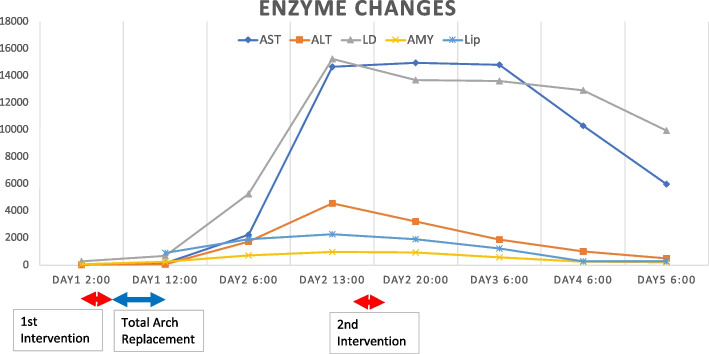
Enzyme changes

**Fig. 3:**
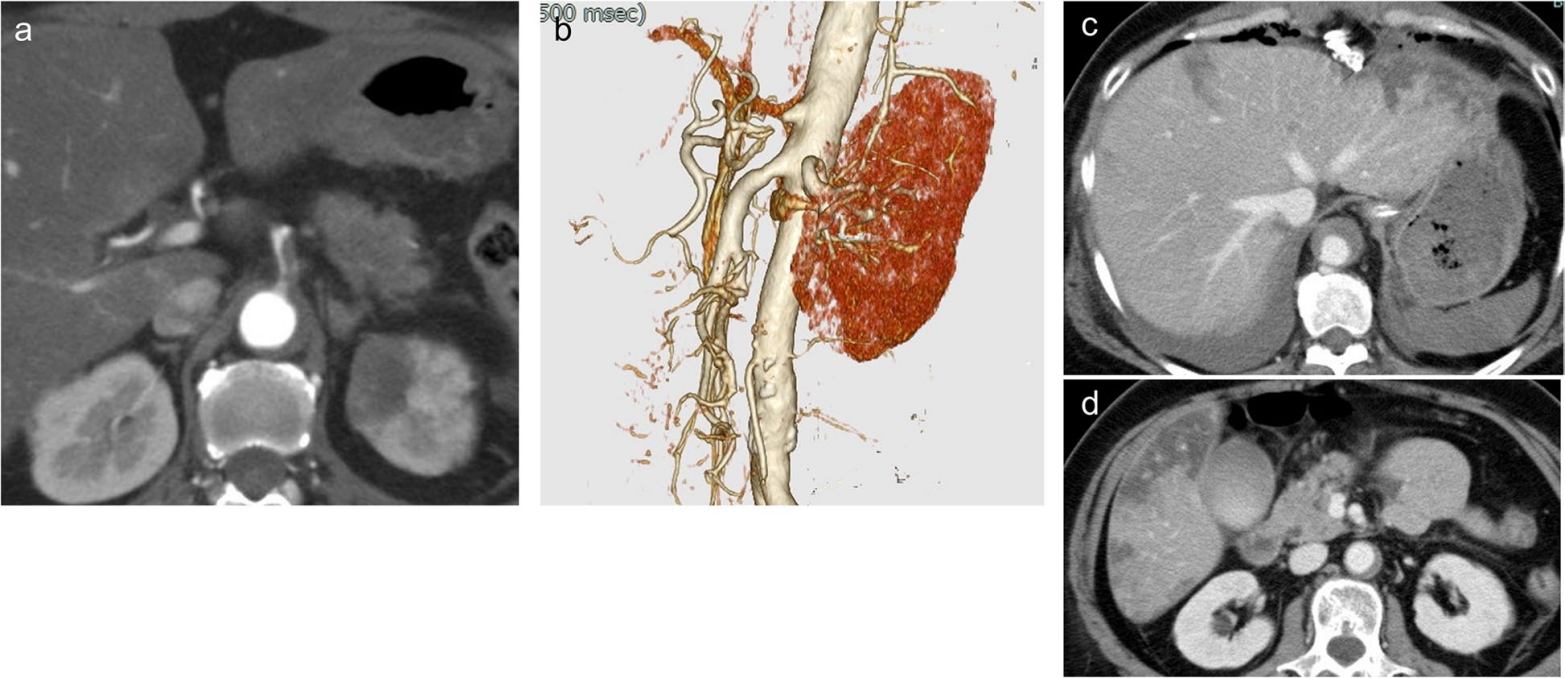
**a**, **b**, **c**, and **d** Postoperative contrast-enhanced CT. **a** and **b** Restenosis of the celiac artery. **c** and **d** An unenhanced liver image on CT

**Fig. 4:**
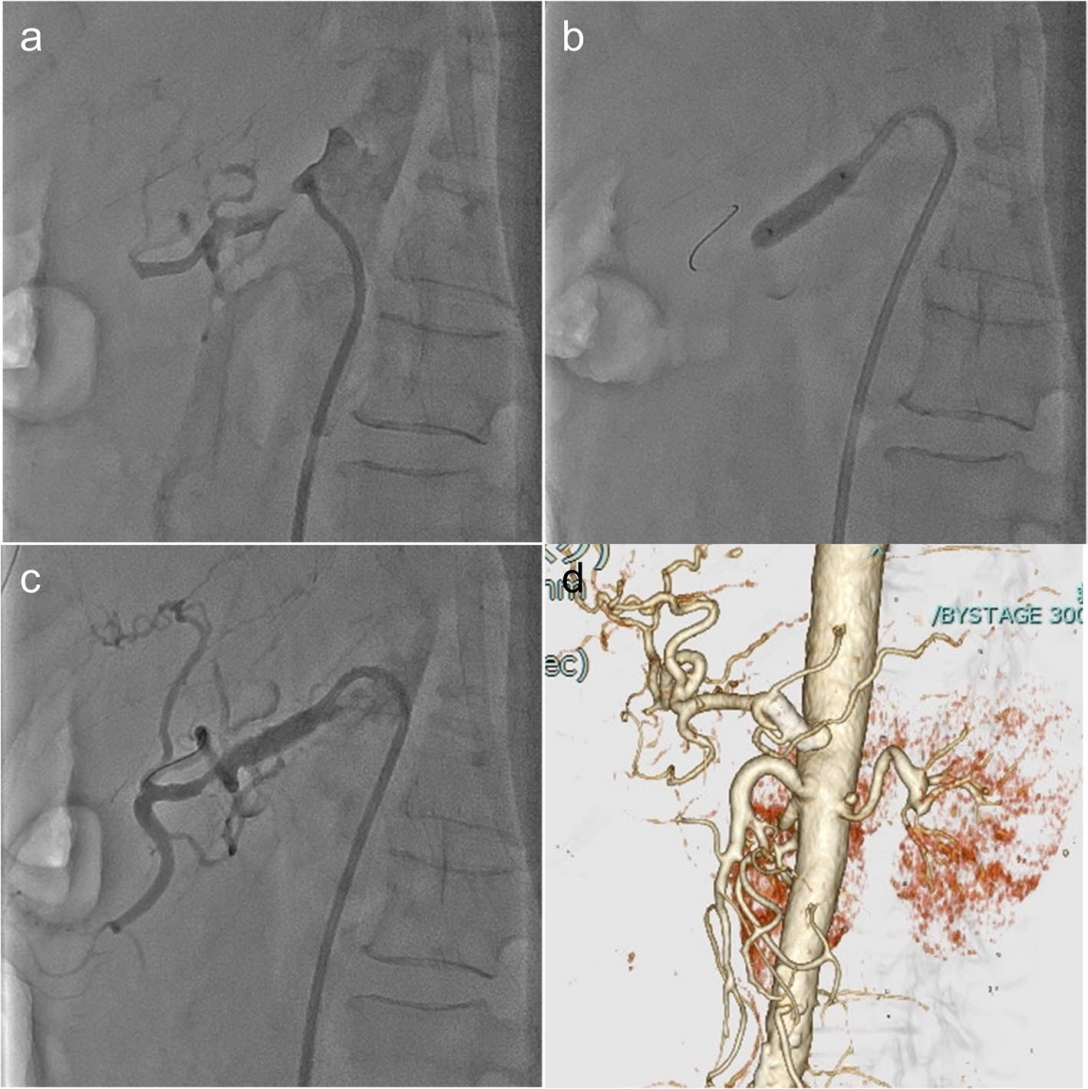
**a**, **b**, **c**, and **d** Second endovascular treatment and final CT findings. **a** The re-occluded celiac artery. **b** A long balloon expandable stent was deployed. **c** Improved blood flow after stenting. **d** CT findings after stenting

Laboratory data obtained immediately following surgery revealed no major abnormalities. However, the day after surgery, laboratory results indicated a marked increase in transaminase, amylase, and lipase levels (Fig. 2). In addition, contrast-enhanced CT revealed restenosis of the celiac artery (Fig. 3a and b) and unenhanced liver image (Fig. 3 c and d). Accordingly, endovascular treatment was repeated, and a 7 mm in diameter and 19-mm-long balloon expandable stent (Omnilink Elite; Abbott Vascular, Santa Clara, CA, USA) was deployed across the severely stenotic origin of the celiac artery (Fig. 4a, b, c, d). Extensive hepatic infarction and pancreatitis occurred but were minimized, and the patient survived. The patient is now in good health 3 years after surgery.

## Discussion and conclusions

Visceral malperfusion, defined as decreased perfusion through the celiac trunk, superior mesenteric artery, and inferior mesenteric artery, is detected in 3.7% of patients with type A acute aortic dissection [1]. In patients with visceral malperfusion, hospital mortality after surgical/hybrid treatment was significantly lower than mortality after medical management and endovascular treatment. Therefore, aggressive surgical/hybrid treatment is necessary in cases of type A acute aortic dissection with visceral malperfusion.

While there is a significant risk associated with visceral malperfusion, ischemia due to occlusion of the celiac artery alone is rare due to the presence of extensive collateral pathways in the mesenteric circulation. Therefore, covering the celiac artery during thoracic endovascular aortic repair is often performed without ischemic complications [2, 3]. However, some cases have been reported where stenting was required for isolated celiac artery occlusion due to type B acute aortic dissection [4, 5]. In these reports, severe abdominal pain and elevated liver enzyme levels were observed, and the collateral pathways between the celiac artery and superior mesenteric artery were found to be inadequate. In these cases, percutaneous transluminal angioplasty was effective.

In our case, CT images similarly showed severe stenosis of celiac artery and no apparent arterial communication between the celiac artery and the superior mesenteric artery (Fig. 1d). These findings and severe abdominal pain may predict upper abdominal organ ischemia in patients with acute celiac malperfusion. Therefore, it should be kept in mind that although acute celiac artery occlusion alone is unlikely to cause visceral ischemic complications, revascularization of the occluded celiac artery should be considered in cases with severe abdominal pain, elevated liver enzymes, and an unenhanced liver image on CT.

We previously reported that an early reperfusion strategy could improve outcomes in cases of malperfusion in type A acute aortic dissection [6]. We usually perform the central repair of type A aortic dissection complicated by visceral malperfusion due to superior mesenteric artery occlusion after direct perfusion of the superior mesenteric artery by laparotomy rather than endovascular treatment. This is because laparotomy allows for a reliable assessment of the presence of intestinal necrosis and the impact of central repair on blood flow. In addition, the treatment can be performed simultaneously without using a hybrid operating room, which minimizes time loss.

This patient had a single malperfusion of the celiac artery, the mechanism of which was static and could not be improved by central repair. Even if an emergency laparotomy was performed, there was no sign of intestinal tract ischemia, and little other information could be obtained. In addition, direct perfusion to the celiac artery region is technically more difficult than that to the superior mesenteric artery. Therefore, we decided to proceed with the endovascular treatment in this case. At that time, there was no appropriately sized stent in stock, so only balloon angioplasty was performed. Angiography revealed good blood flow, and the patient’s abdominal pain improved; therefore, the central repair was performed. Revascularization of the celiac artery with stent insertion before central repair would be better in this case to prevent progressive organ damage. To our knowledge, this is the first report of early reperfusion of an occluded celiac artery before central repair for type A acute aortic dissection.

In conclusion, a single occlusion of the celiac artery complicated by acute aortic dissection can cause upper abdominal organ ischemia. In such cases, early reperfusion should be considered.

## Data Availability

Not applicable.
